# Bioavailability of subcutaneous and intramuscular administrated buprenorphine in New Zealand White rabbits

**DOI:** 10.1186/s12917-020-02618-7

**Published:** 2020-11-11

**Authors:** Raad Askar, Elin Fredriksson, Elin Manell, Mikael Hedeland, Ulf Bondesson, Simon Bate, Lena Olsén, Patricia Hedenqvist

**Affiliations:** 1 Department of Molecular Biosciences, The Wenner-Gren Institute, Stockholm University, Stockholm, Sweden; 2 Department of Clinical Sciences, Swedish University of Agricultural Sciences, PO Box 7054, SE-750 07 Uppsala, Sweden; 3 Department of Medicinal Chemistry, Uppsala University, Uppsala, Sweden; 4 Department of Chemistry, Environment and Feed Hygiene, SVA, National Veterinary Institute, Uppsala, Sweden; 5 CMC Statistics, GlaxoSmithKline Medicines Research Centre, Stevenage, UK

**Keywords:** NZW rabbit, Buprenorphine bioavailability, Administration routes, Opioid pharmacokinetics

## Abstract

**Background:**

Buprenorphine is one of the most used analgesics for postoperative pain in rabbits. The recommended dose in rabbits (0.01–0.05 mg/kg) is the same for intravenous (IV), intramuscular (IM), and subcutaneous (SC) administration, despite lack of pharmacokinetic data. Five male and five female New Zealand White rabbits (mean ± SD body weight 3.1 ± 0.3 kg) were administered 0.05 mg/kg buprenorphine by the IV, IM and SC routes and 0.1 mg/kg by the SC route, in a cross-over design with two-week wash-out periods between treatments. Blood was collected before, and up to 8 h post buprenorphine injection, for determination of serum levels by UPHLC-MS/MS.

**Results:**

The area under the time concentration curve (AUC_0-t_) was lower after SC (398 ± 155 ng/mL/min) than IM (696 ± 168 ng/mL/min, *p* < 0.001) and IV (789 ± 189 ng/mL/min, *p* < 0.001) administration. The maximum serum concentration was lower after SC (2.2 ± 1.4 ng/mL) than after IM (11 ± 3.2 ng/mL) administration (p < 0.001). The bioavailability was lower after SC (50 ± 19%) than after IM (95 ± 21%) administration (*p* = 0.006). The elimination half-life was longer after SC (260 ± 120 min) than after IM (148 ± 26 min, *p* = 0.002) as well as IV (139 ± 33 min) injection (*p* < 0.001). An increase in the SC dose from 0.05 to 0.1 mg/kg resulted in an increase in the area under the time concentration curve of 50% in female (*p* = 0.022) and 165% in male rabbits (*p* < 0.001). The bioavailability did not change in the females (36 ± 14%, *p* = 0.6), whereas it increased in the males (71 ± 23%, *p* = 0.008).

**Conclusions:**

The lower bioavailability of 0.05 mg/kg buprenorphine after SC administration could explain the lack of efficacy seen in clinical pain studies in rabbits, using this route. For immediate pain relief, IV or IM administration is therefore be recommended, whereas SC administration may be useful to sustain analgesic serum levels, once efficient pain relief has been achieved. The current data do not support an increase in dose to compensate for the lower SC bioavailability.

**Supplementary Information:**

The online version contains supplementary material available at 10.1186/s12917-020-02618-7.

## Background

The rabbit is a common pet animal and a common animal model for preclinical studies. In 2016, 350,000 rabbits were used for experimental purposes in the EU [1]. Conditions requiring potent analgesic treatment in rabbits include gastrointestinal disorders and orthopaedic post-operative pain [2, 3]. Buprenorphine is a potent and long acting opioid drug, and the most used analgesic in laboratory rabbits [4]. It is an agonist at the μ-opioid receptor, with approximately 30–40 times the potency, and twice the affinity of morphine [5]. The recommendation for buprenorphine doses in rabbits are the same regardless of administration route; 0.01–0.05 mg/kg IV, SC and IM [2, 6, 7]. There are however few pharmacokinetic studies in rabbits [8–11] and none on the bioavailability after SC or IM administration. Likewise, there are few published studies on the effect of buprenorphine on clinical pain in rabbits. These however indicate that SC administration of recommended doses lack efficacy [11–13], whereas IM and IV administration are reported to have effect [14, 15]. Further, plasma concentrations vary markedly after SC administration of buprenorphine in female New Zealand White (NZW) rabbits [11]. From studies in cats it is known that the bioavailability of recommended doses of SC administered buprenorphine is low [16], and that postoperative pain scores are higher after SC than IM or IV administration of the same dose [17]. The SC dose may result in an inadequate concentration gradient for the buprenorphine to leave the SC tissues, resulting in a smaller than predicted exposure [18].

Pharmacokinetic (PK) data is important for choosing doses and routes of drugs. The aim of the present study was to establish the bioavailability of SC and IM administered buprenorphine in rabbits, and if necessary, revise the current recommendations of administration.

The study was designed to test the hypotheses that a recommended dose of buprenorphine leads to: 1) a lower total exposure over time, as measured by the area under the concentration curve (AUC); 2) lower maximum serum levels (C_max_) after SC than after IM administration and that; 3) a higher dose can compensate for the lower AUC and C_max_ of SC administration.

## Results

All rabbits were clinically healthy and easy to handle. Hematological data were within normal ranges.

### Pharmacokinetics

**Fig. 1:**
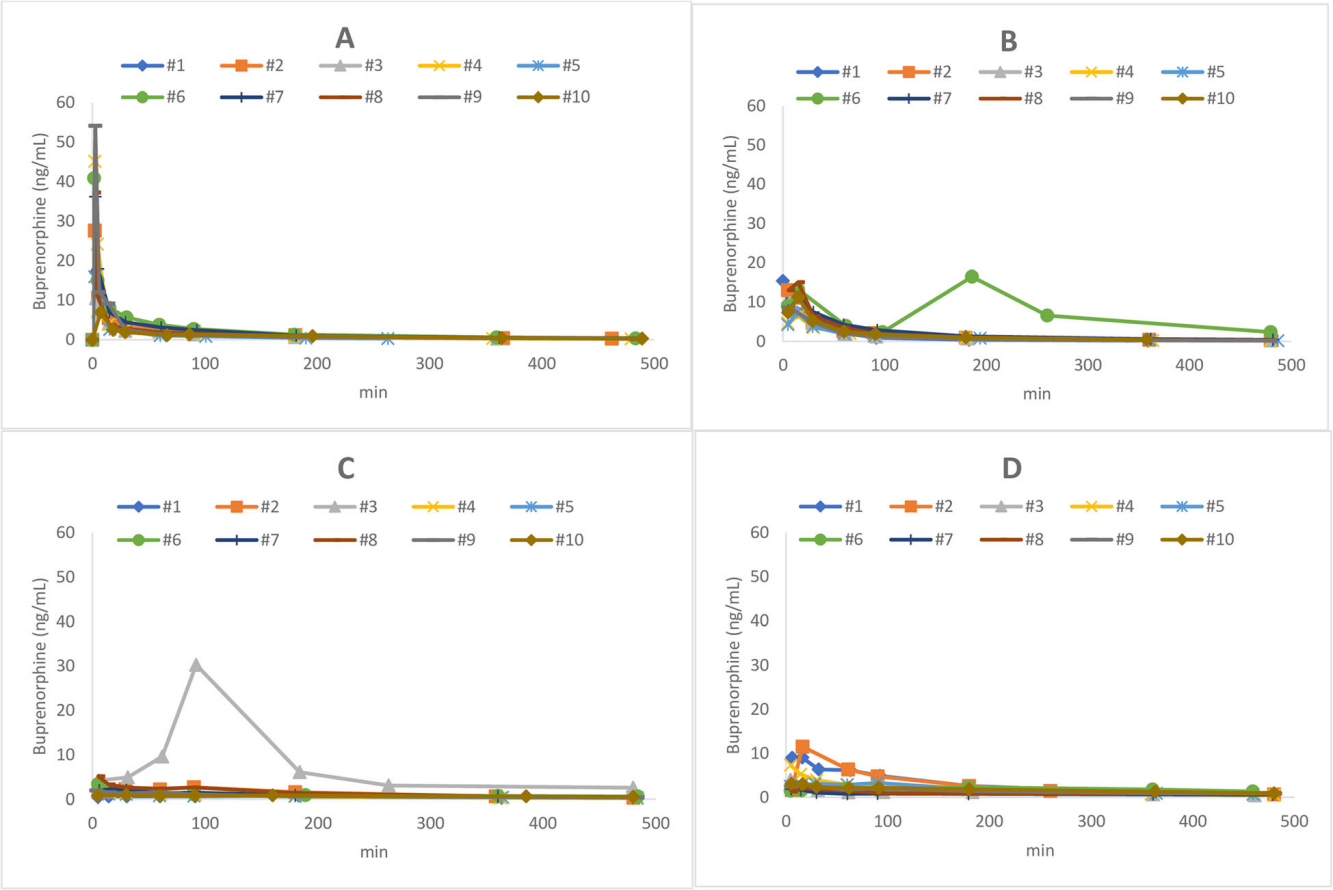
**a**-**d**: Buprenorphine serum concentrations over time in 10 rabbits after administration of 0.05 mg/kg buprenorphine **a**) by the IV route, **b**) by the IM route and **c**) by the SC route and ) after administration of 0.1 mg/kg by the SC route

Individual data on buprenorphine serum concentration over time are shown in Fig. 1a-d. The data from rabbit #6 after 0.05 mg/kg IM and rabbit # 3 after 0.05 mg/kg SC were significantly different from the others and therefore excluded from the data analysis. Buprenorphine mean resident time (MRT) was 180 min ± 54 min, volume of distribution at steady state (Vd_ss_) was 10.2 ± 3.2 L/kg and clearance (CL) 38 ± 11 mL/kg/min.

### Comparison of administration routes (0.05 mg/kg)

**Fig. 2:**
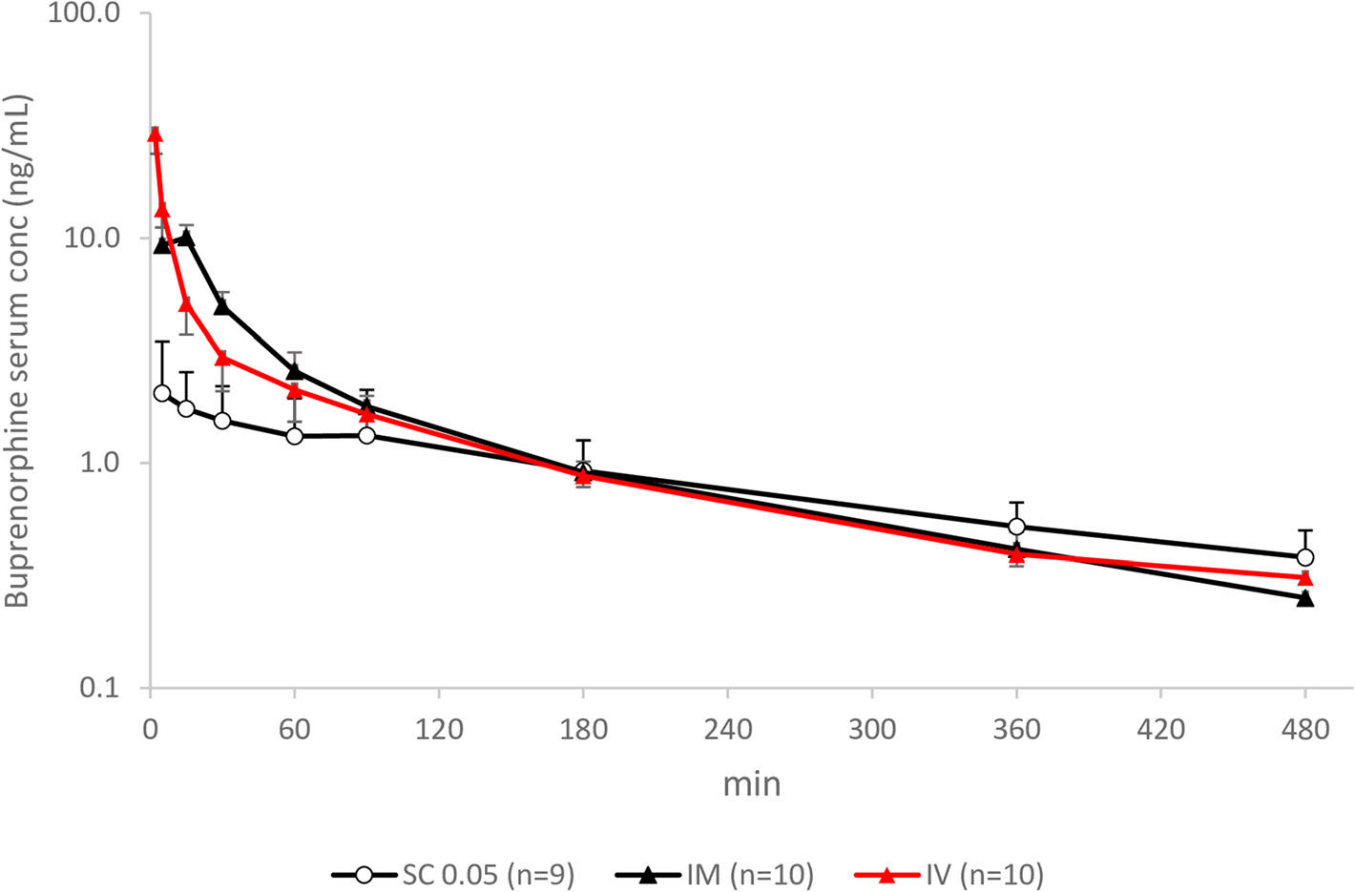
Semilogarithmic graph of buprenorphine serum concentrations in 10 NZW rabbits after administration of 0.05 mg/kg by the IV, IM and SC routes (mean ± SD)

See Table 1 and Fig. 2. The administration route had an overall effect on the AUC_0-t_ (*p* < 0.001), the AUC_0-∞_ (*p* = 0.003), C_max_ (*p* < 0.001), bioavailability (F, *p* = 0.006) and elimination half-life (t_½_, p < 0.001), but not on t_max_ (*p* = 0.379). The AUC_0-t_ and AUC_0-∞_ were higher in females than males (*p* = 0.0495 and *p* = 0.02, respectively): 20% after SC and IM, and 40% higher after IV administration. There was an overall effect of treatment day on C_max_ (*p* = 0.013) and of sex on t_½_ (*p* = 0.041). There was no consistent effect of treatment day across the responses.

**Table 1:** Pharmacokinetic parameters of SC, IM and IV administered buprenorphine in 10 New Zealand White Rabbits

PK parameter	SC 0.05 mg/kg [mean (SD) range]	IM 0.05 mg/kg [mean (SD) range]	IV 0.05 mg/kg [mean (SD) range]
C_max_/C_0_ (ng/mL)	2.2 (1.4) 0.78–5.15	11 (3.2) 6.49–15.4***	71 (44) 18–169
F (%)	50 (19) 26–82	95 (21) 53–113**	–
t_½_ (min)	260 (120) 138–495	148 (26) 113–191**	139 (33) 103–197**
t_max_ (min)	39 (60) 5–180	11 (5) 5–15	–

*SC* subcutaneous, *IM* intramuscular, *IV* intravenous, *C*_*max*_ maximum concentration (SC, IM), *C*_*0*_ calculated maximal concentration at time 0 (IV), *F* bioavailability, *t*_*½*_ elimination half-life, *t*_*max*_ time at maximum concentration. C_max_ was compared between SC and IM routes. (**p < 0.01. ***p < 0.001 two-way ANOVA with route and sex as independent factors and animal and treatment day as blocking factors This was followed by planned comparisons on the predicted means to compare the IV and IM routes back to SC)

**Fig. 3:**
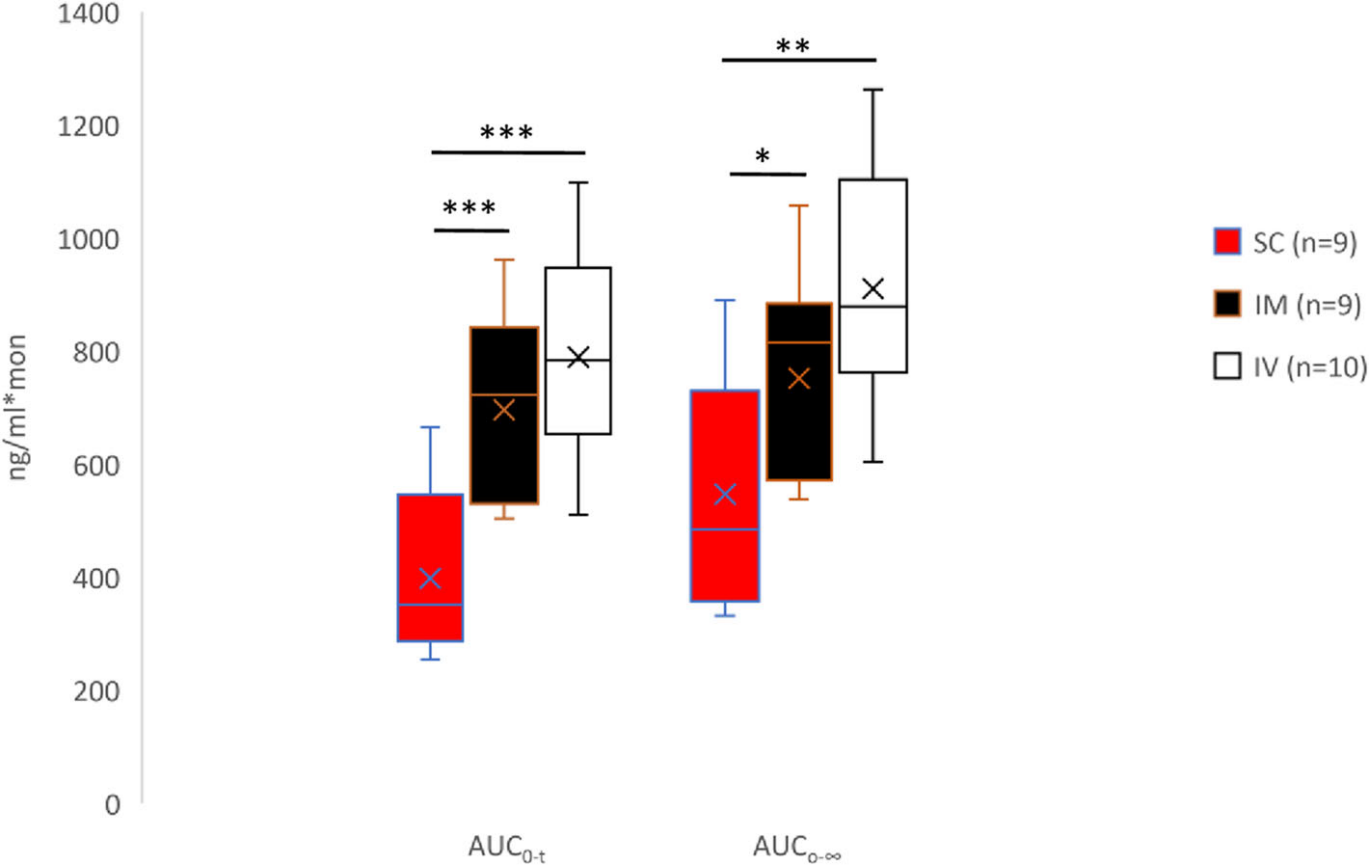
Boxplot of buprenorphine area under the time concentration curves (AUC_0-t_ and AUC_0-∞_) in 10 NZW rabbits after administration of 0.05 mg/kg by the IV, IM and SC routes. Data displayed as minimum, first quartile, median, mean (X), third quartile, and maximum. (***p < 0.001, two-way ANOVA with route and sex as independent factors and Animal and treatment day as blocking factors This was followed by planned comparisons on the predicted means to compare the IV and IM routes back to SC)

**Fig. 4:**
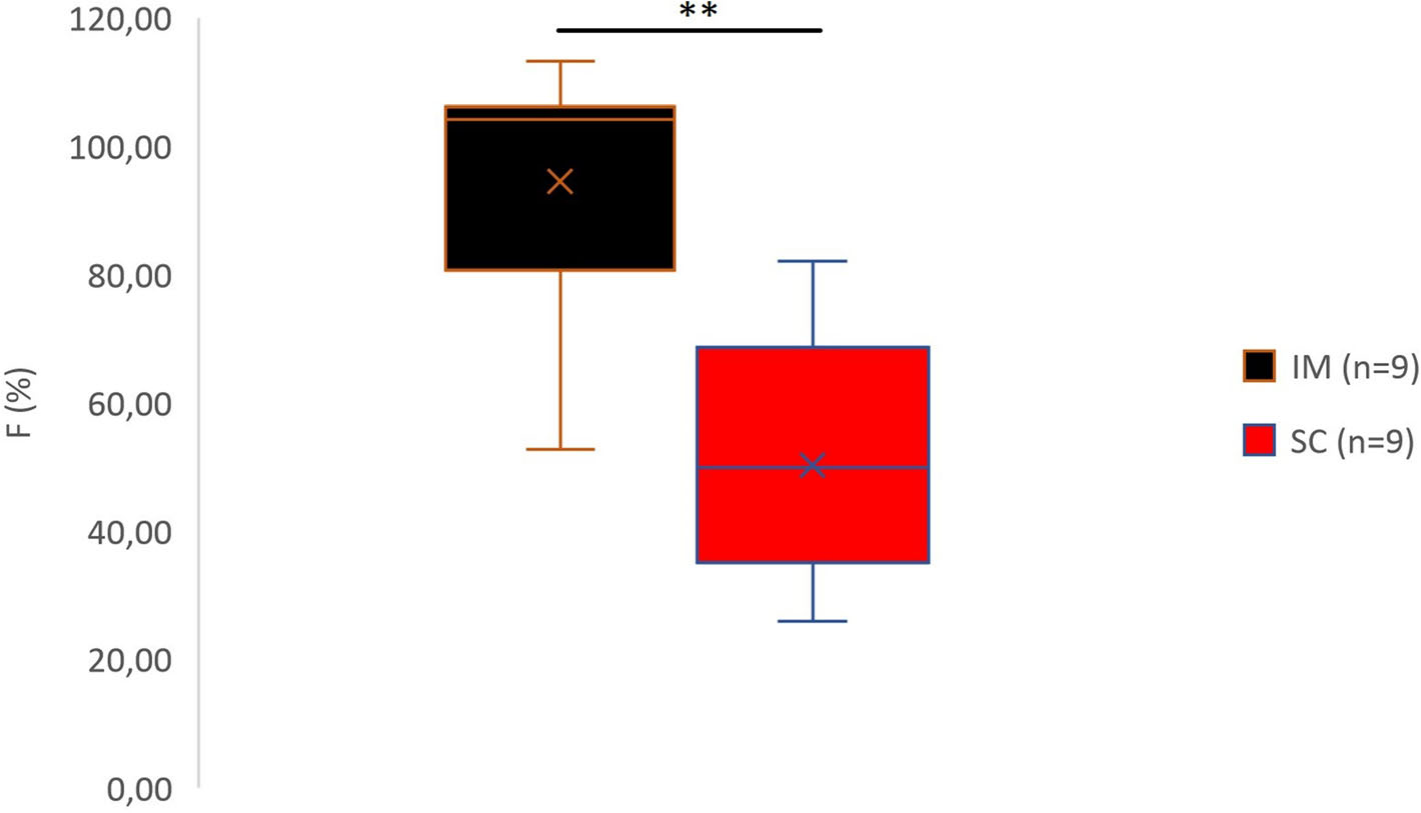
Boxplot of buprenorphine bioavailability (F) in 10 NZW rabbits after administration of 0.05 mg/kg by the IM and SC routes. Data displayed as minimum, first quartile, median, mean (X), third quartile, and maximum. (**p < 0.01, two-way ANOVA with route and sex as independent factors and animal and treatment day as blocking factors This was followed by planned comparisons on the predicted means to compare the IV and IM routes back to SC)

The AUC_0-t_ and AUC_0-∞_ were lower after SC than IM (*p* < 0.001 and *p* = 0.03, respectively) and IV (*p* < 0.001 and *p* = 0.001, respectively) administration (Fig. 3) and C_max_ was lower after SC than IM administration (p < 0.001), as was F (*p* = 0.006 Fig. 4). The t_½_ was longer in females (*p* = 0.041) and longer in SC than IM injection (*p* = 0.002) or IV injection (*p* < 0.001).

**Fig. 5:**
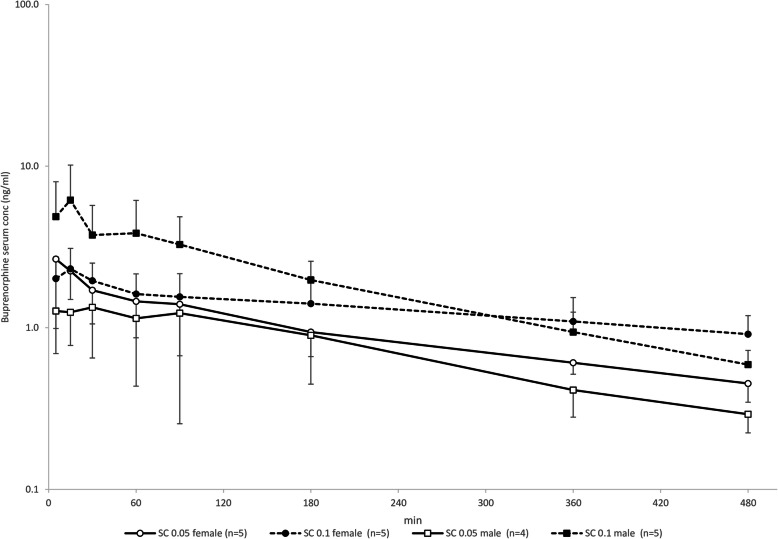
Semilogarithmic graph of buprenorphine serum concentrations in 10 NZW rabbits after administration of 0.05 and 0.1 mg/kg by the subcutaneous (SC) route (mean ± SD)

### Comparison of SC doses (0.05 and 0.1 mg/kg)

See Table 2 and Fig. 5. AUC_0-t_ and AUC_0-∞_ were higher after administration of 0.1 than 0.05 mg/kg SC in females (*p* = 0.022 and *p* < 0.001, respectively) and males (p < 0.001). C_max_ was higher after administration of 0.1 than 0.05 mg/kg SC in males (p < 0.001), but not in females (*p* = 0.150), as was F (*p* = 0.008 and *p* = 0.601, respectively). T_½_ did not differ between 0.1 and 0.05 mg/kg SC in males (*p* = 0.075) or females (*p* = 0.222). T_max_ was longer after administration of 0.05 than 0.1 mg/kg SC in males (*p* = 0.017) but not females (*p* = 0.871).

**Table 2:** Pharmacokinetic parameters of two doses administered SC in 10 New Zealand White Rabbits

PK parameter	Sex	SC 0.05 [mean ± SD (range)]	SC 0.1 [mean ± SD (range)]
AUC_0-t_ (ng/mL/min)	F	423 ± 149 (285–665)	633 ± 211 (362–863)*
	M	366 ± 178 (254–632)	958 ± 387 (470–1357)***
AUC_0-∞_ (ng/mL/min)	F	634 ± 204 (381–890)	1207 ± 261 (961–1572)***
	M	439 ± 180 (332708)	1093 ± 388 (559–1501)***
C_max_ (ng/mL)	F	2.6 ± 1.7 (1.0–5.2)	2.5 ± 0.6 (1.8–3.3)
	M	1.6 ± 0.8 (0.8–2.7)	7.0 ± 3.4 (3.3–11.5)**
Bioavailability (%)	F	48 ± 21 (26–81)	36 ± 14 (20–58)
	M	53 ± 22 (35–82)	71 ± 23 (43–94)**
t_½_ (min)	F	315 ± 139 (156–495)	444 ± 185 (305–717)
	M	192 ± 38 (138–218)	162 ± 31 (130–201)
t_max_ (min)	F	12 ± 11 (5–30)	14 ± 10 (5–30)
	M	73 ± 81 (5–180)	12 ± 11 (5–30)*

*SC* subcutaneous, *AUC*_*0-t*_ area under the concentration curve from t0 to t480min, *AUC*_*0-∞*_ area under the concentration curve from t0 to infinity, *C*_*max*_ maximum concentration, *Bioavailability* AUC_0-t_ IM or SC / AUC_0-t_ IV, *t*_*½*_ elimination half-life, *t*_*max*_ time at maximum concentration. (*p < 0.05, **p < 0.01. ***p < 0.001 two-way ANOVA with route and sex as independent factors, and animal and treatment day as blocking factors, followed by comparison between the SC 0.05 mg/kg and SC 0.1 mg/kg groups)

There was no evidence of an effect of bodyweight on any of the pharmacokinetic parameters, or a consistent effect of treatment day.

## Discussion

Analgesia protocols in rabbits are limited compared with those for cats and dogs, and poor management of pain may be one reason for the high mortality found postoperatively in pet rabbits [19]. There is also a lack of pharmacokinetic data for several analgesic drugs used in rabbits. The current study was aimed at establishing the bioavailability of SC and IM administered buprenorphine in rabbits, because of evidence of poor clinical efficacy after SC administration [11–13]. The results show that the C_max_ and the bioavailability of a 0.05 mg/kg buprenorphine dose was markedly lower after SC than IM administration. The high bioavailability after IM injection is in contrast with other lipophilic drugs in aqueous solutions, which are poorly and erratically absorbed after both SC and IM administration [20]. It is also in contrast with findings in cats, which show an IM bioavailability of 46% of a recommended dose [16]. If drug absorption is slow or irregular, the peak concentrations will be lower and can occur later, as shown in the current study. T_max_ (39 ± 60 min) was long and varied widely between rabbits after SC administration of 0.05 mg/kg. After IM injection, t_max_ was much shorter and less variable (11 ± 5 min). The amount of subcutaneous fat will influence the absorption rate because fatty tissue is less vascularized [20].

The difference in absorption rate may also affect the t_½_, as the drug elimination rate is limited by the drug absorption rate. In the present study the t_½_ was significantly longer after SC than IV and IM injection (*p* < 0.001). Further, the elimination phases of the IV and SC administration routes are not parallel (Fig. 2). This can be an effect of a delay in SC absorption, which in turn prolongs the elimination phase, a so-called flip-flop phenomenon. The prolonged t_½_ can extend the duration of action of a drug, but the terminal exponential phase is usually reached only when plasma drug levels are sub-therapeutic. For this reason, the half-life corresponding to the terminal exponential phase cannot always be used in selecting an appropriate dosing interval. The longest elimination half-life in the current study was 717 min in a female rabbit given the high SC dose. In these cases, the absorption/distribution will play a significant role in the terminal phase of the pharmacokinetic profile and thus explains the over 20% difference in AUC_0-t_ compared to AUC_0-∞_ for the females after SC 0.05 and 0.1 mg/kg. For a more precise calculation of AUC_0-∞_, blood samples at later time points would have been necessary after SC administration.

From previous studies in rabbits, an IV dose six times higher than in the present study (0.3 mg/kg) gave rise to a plasma concentration of > 6 ng/mL for 4 h, with a t_½_ of 233 min [10]. The IV t_½_ in the current study was shorter (160 min). Linhardt et al. [21] administered a three times higher dose (0.15 mg/kg) IV, resulting in an initial concentration of 90 ng/mL at t = 2 min. This is comparable to the 2 min sample in the present study, which was approximately one-third (32 ng/mL). Park et al. [5] administered 0.1 mg/kg SC in 3 male NZW rabbits of unknown age, resulting in C_max_ of 17.5 ng/mL, t_max_ at 15 min and an AUC_0-t_ of 1740 ng/mL/min. In the present study C_max_ was 7 ng/mL, t_max_ 12 min and the AUC_0-t_ 633 ng/mL/min.

With the increase of the SC dose to 0.1 mg/kg, the C_max_ and bioavailability increased in the male, but not the female rabbits. The increase in the males could be explained by a higher concentration gradient for the buprenorphine to leave the SC tissues, resulting in a higher-than-predicted C_max_ and AUC_0-t_ [18]. Why this was not the case in the females, is not clear. A possible explanation could be that females have a larger amount of less vascularized subcutaneous fat, which reduced the absorption rate.

Studies in cats show that the bioavailability of SC administered buprenorphine in recommended doses is very low, individual variability high, and clinical efficacy poor [16, 17, 22]. Cats receiving buprenorphine SC after ovariohysterectomy have higher pain scores than those receiving IM or IV administration, which has led to the recommendation to use IM or IV administration in the acute setting [17]. Recently however, an aqueous high concentration formulation of buprenorphine has been licensed for SC administration in cats in the USA (1.8 mg/mL, Simbadol, Zoetis, Pasippany, NJ). The SC dose is 10 times higher than previously recommended in cats [18, 23], and provides 24 h of antinociception in thermal threshold tests [24]. So far, there are no published data on the clinical efficacy of this formulation.

In line with the published data in cats, the results of the current study may explain why Weaver et al. [12] and Goldschlager et al. [25] did not find an effect on pain related parameters post ovariohysterectomy after SC administration of 0.02–0.03 mg/kg buprenorphine, whereas Cooper et al. [15] did after IM administration of 0.03 mg/kg. Parameters studied were feed consumption, body weight gain, fecal corticosteroids, locomotion and rearing. Likewise, no effect on facial pain scores (eye closure and ear position) were detected after SC administration of 0.05 mg/kg buprenorphine in a study of postoperative orthopedic pain [12]. There may be other explanations why an effect could not be detected, such as a low sensitivity of the assessment method.

Initial high serum levels of buprenorphine will more likely result in effective analgesia. This can be achieved by IV and IM injections. The onset of analgesia is slow even after IV administration (15–30 min), as shown in rabbits in nociceptive tests [26] and peak effect occurs even later (60–120 min). Buprenorphine rapidly crosses the blood brain barrier, but is slow in binding to the μ-opioid receptor [27]. The benefits of buprenorphine are its high potency and affinity to the μ-opioid receptor [5]. Buprenorphine’s potency and slow dissociation from opioid receptors result in low therapeutic doses in animals and humans with concomitantly low plasma concentrations. Buprenorphine has a biphasic dissociation; a rapid 1st-phase dissociation of approximately 50% of μ-receptor bound drug followed by a slow 2nd-phase dissociation. The fact that analgesia of buprenorphine exceeds its pharmacokinetic half-life is believed to be partly due to this 2nd slow dissociation [28].

Even if there is no direct correlation between plasma levels of buprenorphine and degree of analgesia (hysteresis effect), and variability in analgesic effect between individual animals, it is vital to know the bioavailability of different administration routes and doses. SC injections have been preferred because they seem to cause the animal less discomfort and are easier to perform than IM injections. In the case of buprenorphine, this welfare concern may lead to undertreated pain.

A weakness of the current study was that the randomization procedure resulted in an uneven distribution of treatments between treatment days. This was potentially the reason why an effect of treatment day was seen on the C_max_ (*p* = 0.02). On the first treatment day, randomization led to half of the rabbits receiving buprenorphine by the IM route, which had a big influence on the mean C_max_ (8 ng/mL). On the fourth treatment day, half received 0.05 mg/kg buprenorphine by the SC route, which resulted in a much lower mean C_max_ that day (3 ng/mL). A block design would most likely have prevented this effect of treament day. There is however no reason to believe that the day had a true impact on the C_max_. The overall effects of route and SC dose were of such magnitude that the results were deemed reliable. The reason why the two excluded cases showed such high serum concentrations could not be determined. Not only were the concentrations more than 3 SD higher than the mean, but the time-concentration curves did not follow a normal pattern. Further, these rabbits did not show an unusual concentration time curve on any of the other treatment days. The only possible explanation imagined is a contamination of the samples during preparation.

## Conclusions

IV and IM administration of 0.05 mg/kg of buprenorphine cannot be replaced with SC administration, due to a markedly lower peak serum concentration and bioavailability with this route. Increasing the SC dose to compensate for the lower bioavailability leads to unpredictable serum concentrations and can therefore not be recommended. Initial treatment by the IV or IM routes may be followed by SC administration to uphold serum concentrations.

## Methods

### Aim

The aim of the study was to determine the bioavailability of buprenorphine after subcutaneous (SC) and intramuscular (IM) injection in New Zealand White (NZW) rabbits.

### Study design

In this prospective cross-over study, five female and five male NZW rabbits each received four treatments with buprenorphine (Vetergesic vet, 0.3 mg/mL, Orion Pharma, Danderyd, Sweden), with 2-week wash-out periods between treatments. The order in which the animal received the treatments was randomized (Excel, Microsoft cooperation, Redmond, WA, USA). The treatments were 0.05 mg/kg buprenorphine by the IV, IM and SC routes, and 0.1 mg/kg by the SC route (see supplement).

### Animals

The rabbits originated from an SPF breeding colony (Lidköpings kaninfarm, Lidköping, Sweden) free from *Clostridium piriforme, Encephalitozoon cuniculi,* rabbit hemorrhagic disesase, rotavirus, *Pasteurella spp.* and *Bordetella bronciseptica*. They were aged 4–5 months, with a mean ± SD body weight of 3.1 ± 0.3 kg. The females were housed 2 and 3 together and the males singly, in pens of 3 m^2^. Aspen wood chips (Tapvei, Paekna, Estonia) and autoclaved straw (local farm, Uppsala, Sweden) were used for bedding, and the pens were cleaned weekly. The pens were furnished with combined shelters and resting shelves. The room temperature was 19 ± 2 °C, the light:dark cycle 8 h:16 h. The rabbits were fed a restricted amount of a pelleted diet (K1 special, Lantmännen, Stockholm) and had free access to autoclaved hay (local farm) and tap water. The rabbits were acclimatized for two weeks, during which members of the research team took turns in spending 15 min per day per pen to accustom the rabbits to the presence of the members and to handling. Body weight was recorded daily during a week before study begin and at each treatment. Before each treatment, the rabbits underwent clinical examination (visual inspection, auscultation of heart and lungs). Before study begin, blood was collected for hematology.

### Drug administration and sampling

For placements of catheters (Venflon IV PVK, 22G, BD AB, Helsingborg, Sweden), the ears were treated with a local anesthetic cream (EMLA®, AstraZeneca, Södertälje, Sweden). After approximately 30 min, a catheter was placed in the central artery of one ear and on the day that buprenorphine was administered IV, one additional in the lateral ear vein of the contralateral ear. A 2 mL blood sample was collected from the arterial catheter before and at each sampling timepoint after administration of buprenorphine. Timepoints for sampling were 2 min (after IV administration only), 5, 10, 15, 30, 60, 90, 180, 360 and 480 min after injection. The time points were based on previous studies. Prior to blood collection, 0.2 mL of blood was discarded and after collection the catheter was flushed slowly with 2 mL of NaCl (Infusion solution, 9 mg/mL, B. Braun, Danderyd, Sweden) and 0.1 mL of heparinized saline (100 IU /mL, Heparin LEO 5000 IU/mL, LEO Pharma, Malmö, Sweden) deposited in the catheter. A maximum volume 7 mL blood/ kg was collected at each treatment. Depending on the weight of the rabbit, and the treatment, a random sample timepoint was sometimes excluded in order not to exceed the recommended maximum blood volume [15% of blood volume/14 d [29]]. Blood was collected in 2 mL serum tubes and left to coagulate at room temperature for at least 60 min. After centrifugation at 3000 g for 15 min, serum was separated and stored at minus 80 °C until analyzed. At the end of each treatment, 4 mg/kg carprofen (Norocarp vet, 50 mg / mL, N-Vet AB, Uppsala, Sweden) was administered SC for 24 h of post procedural analgesia. After the study end, the rabbis were re-used in a non-recovery anaesthesia study euthanized by injection of pentobarbital (Allfatal) IV.

### Buprenorphine analysis

Buprenorphine was quantified in rabbit plasma using ultra-performance liquid chromatography-tandem mass spectrometry (UHPLC-MS/MS) using an Acquity UPLC coupled to a TQS micro tandem mass spectrometer (both Waters Corp, Milford, MA, USA) using an electrospray interface operating in the positive mode. To the plasma samples (250 μL), 50 μL of water, 50 μL of internal standard solution (buprenorphine-d4 40 ng/mL) and 250 μL of sodium carbonate buffer (0.05 M; pH 9.9) were added. Liquid-liquid extraction was performed to 3.0 mL of ter-butyl-methylether for 10 mins. The tubes were centrifuged and frozen at − 70 °C, after which the organic phases were poured into new tubes for evaporation to dryness under nitrogen at 50 °C. The residues were reconstituted in 100 μL of acetonitrile/water/formic acid (9/1/0.01, v/v). The extracts were transferred to vials for UHPLC-MS/MS injection. The column used was an Acquity UPLC BEH C18 (100 × 2.1 mm length x inner diameter, 1.7 μm particle size) from Waters Corp. The mobile phase consisted of (A) 0.1% formic acid in water and (B) 0.1% formic acid in acetonitrile. Gradient elution was performed: initially 15% B for 0.50 min, linear increase to 90% B for 1.0 min, constant at 90% B for 0.50 min, back to 15% B in 0.10 min and constant at 15% B for 0.40 min. The total run time was 2.5 min. The flowrate was 0.40 mL/min and the injection volume was 3.0 μL.

The quantification was performed in the Selective Reaction Monitoring mode (SRM) with the transitions 468 > 396 for buprenorphine [M + H] + (collision energy 36 eV), and 472 > 400 for buprenorphine-d4 [M + H] + (collision energy 38 eV). For quantification, calibrator samples were prepared by spiking standard solutions of the analyte to blank plasma which were analyzed according to the method. The calibration curve was constructed by linear curve fitting using the chromatographic peak area ratio (analyte/internal standard) as a function of analyte concentration with a weighting factor of 1/x. The measurement interval was 0.01 ng/mL – 100 ng/mL. The precision and accuracy of the method were within the acceptance criteria [30].

### Pharmacokinetic analysis

For each animal, route and dose the concentration of buprenorphine were plotted vs. time. Different models and weighting factors were assessed by visual inspection of the curve fits and the residuals’ scatter plots, together with the accuracy of fit measures incorporated in the software (e.g. the Akaike criteria). A non-compartmental model was used to compare all routes. The maximal concentration of buprenorphine in serum (C_max_), t_max_ (time to reach C_max_), t_½_ (half-life) and AUC (area under the plasma concentration curve) were calculated with a non-compartmental model using the PK Solver add-in for Microsoft Office Excel. For IV administration also a 3-compartment model was used to calculate the maximal concentration at time 0/at the time for administration (C_0_).

The bioavailability (F%) for the SC and IM administration routes were calculated from the AUC_0-t_ by using the equation:

$$ Fadm\ \left(\%\right)=100\ x\ \left( AUC adm\ x\  dose\  iv\right)/\left( AUC\  iv\ x\  dose\  adm\right) $$

### Statistical analysis

InVivoStat (Version 4.0) was used for the sample size assessment [31]. The number of rabbits used was selected based on a power calculation, with a significance level set at 5%, and a power of at least 80% to detect at least a 25% change from the area under the time-concentration curve (AUC_0-t_) after IV administration. Data from two animals were removed from the statistical analyses (#3 0.05 mg/kg SC and #6 0.05 mg/kg IM) because the concentration curves did not follow a normal pattern and the concentrations were in part so high than it is unlikely a normal biological variation. All data are included in the supplement data file.

Data were analyzed in two stages. In order to test the overall effect of the administration routes, the data from the 0.05 mg/kg groups were compared by two-way ANOVA with Route and sex as independent factors and animal and treatment day (of administration) as blocking factors (SAS/STAT software, Version 9.4 of the SAS System for Windows 10). This was followed by planned comparisons of the predicted means to compare the IV and IM routes back to SC. In the second stage the additional SC 0.1 mg/kg group was included to allow a comparison between the SC 0.05 mg/kg and SC 0.1 mg/kg groups.

Data are presented as mean ± SD. The level of statistical significance was set to *p* < 0.05.

## Supplementary Information

**Additional file 1.**

**Additional file 2.**

## Data Availability

The raw data is provided in a supplementary file.

## Abbreviations

SC: Subcutaneous
IM: Intramuscular
IV: Intravenous
AUC_0-t_: Area under the curve from t0 to t480 min (last time point sampled)
AUC_0-∞_: Area under the curve from t0 to infinity
t_max_: Time of maximum concentration
C_max_: Maximum concentration
C_0_: Concentration at time point 0
t_½_: Elimination half life
F: Bioavailability
NZW: New Zealand White
