# Correction to: Bioavailability of subcutaneous and intramuscular administrated buprenorphine in New Zealand White rabbits

**DOI:** 10.1186/s12917-021-02858-1

**Published:** 2021-04-16

**Authors:** Raad Askar, Elin Fredriksson, Elin Manell, Mikael Hedeland, Ulf Bondesson, Simon Bate, Lena Olsén, Patricia Hedenqvist

**Affiliations:** 1 Department of Molecular Biosciences, The Wenner-Gren Institute, Stockholm University, Stockholm, Sweden; 2 Department of Clinical Sciences, Swedish University of Agricultural Sciences, PO Box 7054, SE-750 07 Uppsala, Sweden; 3 Department of Medicinal Chemistry, Uppsala University, Uppsala, Sweden; 4 Department of Chemistry, Environment and Feed Hygiene, SVA, National Veterinary Institute, Uppsala, Sweden; 5 CMC Statistics, GlaxoSmithKline Medicines Research Centre, Stevenage, UK

**Correction to: BMC Vet Res. 16, 436 (2020)**

**https://doi.org/10.1186/s12917-020-02618-7**

The original article [1] contained an error in the naming of the boxplots in Fig. 4. This has since been corrected.
