# Supplementary material for: Bioavailability of subcutaneous and intramuscular administrated buprenorphine in New Zealand White rabbits
Source: BMC Vet Res. 2020 Nov 11;16:436. doi: 10.1186/s12917-020-02618-7 (PMC7656698; doi:10.1186/s12917-020-02618-7)
Supplement: Supplementary file 2 — Additional file 2. [file 12917_2020_2618_MOESM2_ESM.docx]

Table of administration routes and doses of buprenorphine in mg/kg in 10 New Zealand Rabbits. A two-week wash-out period was left between each treatment.

| Subject | Treatment 1 | Treatment 2 | Treatment 3 | Treatment 4 |
| --- | --- | --- | --- | --- |
| 1 | IM 0.05 | SC 0.1 | IV 0.05 | SC 0.05 |
| 2 | SC 0.1 | IM 0.05 | SC 0.05 | IV 0.05 |
| 3 | SC 0.05 | IV 0.05 | IM 0.05 | SC 0.1 |
| 4 | IM 0.05 | SC 0.05 | IV 0.05 | SC 0.1 |
| 5 | IV 0.05 | IM 0.05 | SC 0.05 | SC 0.1 |
| 6 | IM 0.05 | SC 0.05 | IV 0.05 | SC 0.1 |
| 7 | IM 0.05 | SC 0.05 | IV 0.05 | SC 0.1 |
| 8 | SC 0.1 | IM 0.05 | SC 0.05 | IV 0.05 |
| 9 | IM 0.05 | SC 0.05 | SC 0.1 | IV 0.05 |
| 10 | SC 0.05 | SC 0.1 | IM 0.05 | IV 0.05 |

IM=intramuscular, SC= subcutaneous, IV=intravenous administration
